# Challenges and opportunities for defining the role and value of meat for our global society and economy

**DOI:** 10.1093/af/vfad002

**Published:** 2023-04-15

**Authors:** Rod Polkinghorne, Mohammad Koohmaraie, Collette Kaster, Declan Troy, Andrea Rosati

**Affiliations:** Birkenwood Pty Ltd, Blandford, Australia; Meat Division, IEH Laboratories and Consulting Group, Lake Forest Park, WA, United States; American Meat Science Association, Kearney, MO,United States; Teagasc Food Research Centre, Teagasc – The Irish Agricultural and Food Development Authority, Dublin, Ireland; EAAP, European Federation of Animal Science, Terni, Italy

**Keywords:** anti-meat rhetoric, collaboration, industry engagement, research engagement

**Implications**

- There is currently a significant need to develop future leaders and scientists across the meat industry, encompassing enterprises from livestock to commercial meat product production, research organizations, government, industry bodies, and educational institutions as well as funding for effective research on meat production and products. New models are needed to counter the reduction in traditional delivery through postsecondary education, especially at facilities that also conduct research. An essential element should be greater collaboration at institutional, government, country, and global levels which will demand open sharing of resources and expertise.
- Timely access and early commercial application of scientific advances to improve productivity, human dietary, and environmental outcomes help to supplant the pursuit of controlling intellectual property and related revenue, a return to more open science and shared resources. A careful study of the issues discussed here reveals that two major changes are required: 1) Greater involvement of large and small commercial meat business enterprises (beneficiaries of much of the research outcome), in investing/funding and 2) to maintain and increase industry’s involvement, scientists need to deliver timely solutions.
- To ensure viability and create research opportunities, new models for development and delivery of science that advances the mission to improve the production of high quality, safe, nutritious, and affordable meat will need to diverge from the historic “silos” of individual disciplines and evolve to highly collaborative and integrated arrangements where various sciences embrace their common base and are enriched by the mutual contact. Cooperative structures that encourage active collaboration and targeted funding across research, educational, government, and commercial groups need to become the norm as the traditional model of meat science delivered through large government-funded institutions has almost passed. The industry will need to increase engagement and scientists will need to collaborate across disciplines to deliver tangible results to maintain critical mass and ensure that livestock industries are able to continue their path forward based on scientific and technological improvement for the benefit of society.
- Lack of—or reduction of—sustainable funding has contributed to a reduction in young scientists choosing meat science as a career, and competition for limited and shrinking funds has resulted in reduced collaborative problem-solving projects and more salary competition between business and research-related roles. Industry identifies talented researchers and often leverages their skill for research management into business management. This funding reduction began decades ago, and the trajectory is not promising. Resources are required to robustly interact with nutritional guidelines, environmental challenges including measurement and accounting systems for continual improvement in animal welfare outcomes and to provide production models adapted to Africa and the global south.
-  Everybody eats! This fundamental truth combined with the outstanding nutritional properties of animal-based foods directly establishes the case for meat science to be taught in conjunction with human nutrition, food science, and medical disciplines. This aligns with many of the contemporary meat science priorities driven by an emphasis on environmental impact (e.g., methane reduction technologies, new packaging systems to replace plastics, water use), eating quality (e.g., flavor chemistry, consumer sensory impact), deeper understanding of animal welfare and stress (including measurement and remediation), and the rapidly expanding interaction with objective technologies to predict, measure, or mechanically process carcasses and cuts.
- We hope this document will stimulate dialogue and encourage the widespread involvement of those who care about animal agriculture and its societal role to address problems and provide long-lasting solutions before it is too late.

## Introduction

There is no doubt that the role of meat in society is being robustly challenged. This era of the challenge has evolved while meat and animal science resources, both facilities and humans, have substantially declined (Cross, 2017, 2022). Examples include the demise of MIRINZ in New Zealand, the CSIRO Cannon Hill facility in Australia, and Bristol in the United Kingdom. Even if science strongly supports the critical role of animals in environmental stewardship (elsewhere in this issue; Thompson et al., 2023) and of meat for human nutrition (elsewhere in this issue; Leroy et al., 2023) and health (elsewhere in this issue; Johnston et al., 2023), alternative views and competing claims have been widely disseminated and influenced societal beliefs. These developments were foreseen almost 30 years ago by Blaxter (1991) who prophetically stated, “in the years to come I am of the opinion that policies will be adumbrated by various pressure groups on the basis of little evidence, pursued assiduously by them, and eventually accepted on the basis that, although not scientifically defensible, they have become politically expedient.” Our response would seem to have been far too slow and far too little as the advent of social media surpassed the reach of peer-reviewed scientific publications.

It is critical that decisions and policies be based on evidence rather than ideology. The scientific community should strive for the highest standards of evidence-based research and provide results accessible to a contemporary nonscientific audience. Effective, transparent, and honest communication is critical, both in presenting evidence and in projecting the livestock and meat industries as offering dynamic, exciting career paths that attract the best and brightest who are engaged by the opportunity to contribute to improved environmental and societal outcomes. We all eat: the industry is of fundamental importance.

## Challenges

A report (Polkinghorne et al., 2022) presenting the findings of an extensive global survey of meat scientists and industry management revealed that societal concerns relating to meat production and consumption were rated highly as a major challenge, requiring at least equal attention as the core muscle and biology science base. These concerns were endorsed during a discussion at the American Meat Science Association’s Reciprocal Meat Conference (**RMC**) and International Congress of Meat Science (**ICoMST**) forums in 2021 and 2022.

Despite the extraordinary economic importance of meat production in developed economies (elsewhere in this issue, Ederer et al., 2023) and critical nutritional contribution to global human health (elsewhere in this issue; Leroy et al., 2023), calls for dramatic reduction in livestock and meat production are often associated with assertions that plant and cell-based foods can and will adequately replace meat and milk in sustainably feeding a human population of 10 billion (Tubb and Seba, 2021). Both mass media and policy makers reference publications in high-ranking scientific journals, often including a long list of authors from high profile institutions (e.g., Willett et al., 2019), to make an unbalanced case against meat, presenting it as an unhealthy food choice. Even if such studies often make use of unsubstantiated assumptions that have been challenged based on the evidence (e.g., Johnston et al., 2019; Zagmutt et al., 2019; Stanton et al., 2022), as also outlined elsewhere in this issue (Johnston et al., 2023), it is often difficult to publish results that deviate from the institutionalized narrative. Doing so may even result in strong personal attacks on authors (Rubin, 2020; Flegal, 2021). In an era where ideology and food politics dominate discussion and are of most concern impacting impartial scientific publication, the need for objectivity-seeking scientific evidence has never been greater.

In addition to the nutritional debate, the anti-meat rhetoric has extended to environmental debates with ruminant livestock challenged on gross greenhouse gas emissions, mostly without reference to net emissions and the potential for these to be at least partially offset due to the short-term methane cycle and recycling of carbon through grazed pastures. As argued elsewhere in this issue (Thompson et al., 2023), these claims also ignore—among many other points of interest—the massive number of wild ruminants and their contribution to the natural carbon cycle over millennia, the historical and current impact of grazing on soil structure and fertility, the potential to valorize marginal lands that are otherwise not suitable for food production, and the unique ability of livestock (especially ruminants) to produce nutrient-dense food from nonhuman edible byproducts and plant material.

The increased rhetoric around these challenges to meat production and consumption has been paralleled by a dramatic decline in resources (Blaxter, 1991; Cross, 2017, 2022) to accomplish necessary research: human, financial, and facilities, across many countries. This includes the removal or significant downsizing of previously prestigious specialist institutions and substantial reduction in public funding allocated toward use in meat product research, with the closing after removal of government funding for the Meat Research Institute (Bristol, United Kingdom) in 1990 and that of MIRINZ in New Zealand pertinent examples. Such evolution presents real concerns as to the ability of the meat research community to adequately support an industry that relies on a strong scientific base to drive innovation and continuing improvement. These programs have delivered improved production efficiency, safety, and quality of traditional meat products in the context of improving animal welfare, animal health, and environmental sustainability.

Challenges relate to the critical fundamental science base capability and capacity in addition to directly applied activity and effective extension of scientific outcomes. Research capabilities have been reduced and scientists are obligated to find their own funding as well as complete an increasing number of noncore tasks increasingly required by the academic system. Thus because of increased workload due to staffing reductions, the requirement to secure their own funding, and a less competitive pay rate, many bright researchers choose to work in applied fields and in industry business roles rather than in traditional research settings. Solutions to counter these challenges and reset the narrative are critical.

## Solutions

Everybody eats! This simple truth logically positions meat, eggs, and dairy at the center of discussion and scientific endeavor relating to human diet and health, further emphasized by the projected need to feed an extra 2 billion people, mostly located in Africa and Southeast Asia, by 2050 (UN, 2022). Global meat production is forecast to reach 373Mt by 2030 (OECD-FAO, 2021). Given the nutrient density and balance of animal products, and their evolutionary role in human development, animal products can be viewed as an essential resource to prevent malnutrition and starvation in regions where food security is already a critical concern (Ederer et al., 2023; Leroy et al., 2023). Furthermore, a substitution of natural animal foods for nonanimal based ultra-processed high-calorie, high carbohydrate, and low-nutritional value products associated with adverse health outcomes in the global north would deliver significant benefits (Scrinis, 2020).

Similarly, and often presented in an African context (Gerber and FOA, 2013; Hillenbrand, 2019), the effective use of grazing animals has been associated with improved environmental conditions in soil health, water retention, and drought resilience (Rowntree, 2020). Conversely, the claims of environmental damage from animals need to be evaluated from evidence and positively addressed based on improving both global food security and environmental outcomes. The important societal role of animal agriculture in both developed and subsistence agricultural systems is also of major human importance (FAO, 2018; Nordhagen, 2020) and is interlinked with health, environmental, economic, and social considerations (Leroy, 2020).

These factors imply that animal related disciplines including animal, veterinary, and meat science should be viewed as essential integrated and fundamental components of other biologically based disciplines, most critically human medicine, nutrition, and environmental studies. In contrast to the isolated “silos” of individual disciplines an interactive environment and common delivery across medicine, nutrition, animal, and meat science of the base biological ABC (Anatomy, Biology, and Chemistry) could accelerate scientific advancement and greatly improve coordination and cooperation across disciplines.

These linkages also logically exist across nonbiological areas including engineering and information technology which are essential and critical components of meat processing systems. The challenges of adapting engineering solutions such as robotics to successfully manage a highly variable natural and perishable raw material and “de-manufacturing” process of reducing carcasses and carcass components to final products, relative to traditional manufacturing of assembling standard size, shape, and texture products provide exciting research opportunities for the most motivated young engineers. The essential understanding of both the biological and engineering attributes could be empowered by facilitating creation of joint teams of meat scientists and engineers and interaction between the disciplines within degree and higher-level programs. The current ideological challenges to the role of meat in society should be alert to the need to engage strongly and in harmony with those in ethics and social science areas.

The extensive global reduction of teaching and research resources should also drive increased collaboration across institutions and institutional locations. Critical high-quality research teams should be built across institutional and global boundaries so that expertise and specialist resources can achieve sufficient mass to deliver efficient high-quality research within shorter timeframes. These collaborations need to be accelerated and driven by the urgent need for industry-wide innovation, thus the accumulated value dominates consideration of IP protection or ownership.

It is critical that fundamental and applied research be strongly and effectively balanced and connected through research institutions and industry employed researchers to both maintain delivery of fundamental research, that is likely to empower yet unidentified solutions or opportunities and applied research to directly address existing industry problems and priorities. The industry is likely to benefit, value, encourage, and most importantly, invest far more than they have in the past in research that will drive innovation and continued improvement, and research teams must fully understand and effectively address industry challenges. While businesses may not fully understand the essential role and process of fundamental pure research and teaching institutions and research scientists may not comprehend the detailed commercial challenges of marketing a huge array of differentiated perishable products from blood and bones to retail meat in proportion to a purchased animal, both need to appreciate the complexity and expertise required and facilitate mutual support for common good.

Funding and individual remuneration systems need to also encourage constructive responses. To this end, academic evaluation criteria need to balance peer-reviewed publication with teaching excellence and commercial impact. High-quality fundamental academic research-motivated students and highly effective industry collaboration are of equal importance and best delivered by individuals with complimentary strengths. Sustainable government, industry, and private funding mechanisms require alignment with the balance of needs to ensure human resources for industry viability which may be enabled by related and integrated educational and research arrangements. These arrangements can reflect increased collaborative activity within and across institutions, across disciplines and between institutions, industry, and government policy. They can also extend over significant decades and long timeframes to enable secure career paths and the accumulation of specialist knowledge and expertise. Furthermore, university overhead charges and conditions applied on collaborative research projects need to recognize the value of attracting external stakeholders rather than acting as a deterrent.

Clearly, the consumer, through commercial application, is the beneficiary of most of the scientific discoveries which sustains the argument for extensive ongoing government investment. However, the forecast for governments to be the primary sponsor of meat science research is not promising given the increasing pressure on government expenditures limiting their capacity to continue to be the primary sponsor of meat science research conducted within businesses, at educational institutions, or through entirely government-funded research facilities.

Finally, but of critical importance, both science and industry need to adopt more effective and coordinated communication approaches to explain, justify, and promote the role of animals and animal-sourced foods in sustaining and improving environmental outcomes, animal and human health and in enriching society. Livestock and meat industry organizations should also be cognizant of the need to ensure they reach a consensus prior to advocating federal and local government policy. Interactions regarding food and its sources require initiation at a young age and continue with an allied commitment to nonbiased and honest, transparent communication.

## Prospective Models

New models of communication, education, research, and production operations have always evolved over time. The accelerated rate of change and coordinated attack on meat production and consumption over recent decades, however, demands an immediate response to ensure the essential role of animals and animal-sourced foods is reinforced. Further, new collaborative organizational models are essential to address the decline in human and physical educational and research resources. This is essential to ensure industry can be supported through a particularly challenging period of global climate change and predicted human population growth. This requires new thinking, embracing of change, and a broad collaborative global philosophy contrary to the competitive structures of the past that often encouraged isolated and competitive individual activity. Some useful successful models exist, and more should evolve.

As an example, the Queens University Belfast (**QUB**) structure has the Faculty of Medicine, Health and Life Sciences branching down to the Institute for Global Food Security and the School of Biological Sciences that provides Agriculture, Food and Veterinary research and teaching. Companion Schools of Medicine, Pharmacy, and Nursing ensure linkage under a philosophy that Agriculture and Food deliver health with Medicine addressing sickness. These associations deserve wider consideration. The UK structure of academic remuneration and evaluation having a set weighting for impact (moved from 5% to 45% over the past decade or so) in balance with peer-reviewed papers has been seen to stimulate industry engagement and could be a useful model. The appointment of Professors of Practice within QUB also reflects the desire to engage and value practical commercial experience in addition to an academic qualification.

The QUB model contrasts with the Australian experience, where Universities place very low value on extension and outreach for remuneration and promotion practices. The CSIRO meat research commitment and traditionally strong State Government agricultural departments, which once played a major role in extension activity in addition to managing research farms and facilities, have been dramatically scaled back and defunded. While private consultants have expanded their role in delivery of technical information to businesses, this model is seen as less accessible than the prior arrangements and often relies on substantial support from Meat and Livestock Australia (**MLA**) or the Australian Meat Processor Corporation (**AMPC**). However, essential extension funding by these bodies to offset reductions in state-funded activity reduces the capacity for research investment.

Research funding varies widely across countries with voluntary or fixed statutory levies, a feature of the Australian, U.S., and other industries. The Australian beef levy is currently set at AU$5.00 per head of which $0.92 per transaction for grass fed and $1.50 for grain fed cattle is allocated to Research and Development (R&D). The R&D levy component is then matched by federal government funding. Additional matching federal funding is available for approved individual company project funding. Providing the projects have value to the Australian industry, they can also be accessed by international groups. The U.S. Beef Checkoff program has similar objectives directed at marketing, education, and research activity but only collects funds from livestock producers. Other agencies including the United States Department of Agriculture (**USDA**) also invest in American research, but priorities have evolved and the funding for traditional meat research has declined. The United States does have impressive early life livestock and meat engagement achieved through 4-H, FFA (formerly the Future Farmers of America), and intercollegiate meat judging competitions. The American Meat Science Association (**AMSA**) is also proactive in engaging with postsecondary animal and meat science students at an early career level through workshops, mentoring, scholarships, internships, and the annual RMC.

Within the US philanthropic grants also provide extensive valuable funding for facilities and operational funding directly or with matching funding from other sources, the Texas Government Texas Research Incentive Program (**TRIP**) a good example, providing up to 100% matching funding for gift donations over US$1 million. The FFAR also provides similar funding possibilities. While these funds can provide valuable supplemental funding, they cannot be relied on for ongoing program development.

Other large-scale programs designed to stimulate innovation and collaboration include the Meat Technology Ireland (**MTI**) in Ireland and Cooperative Research Centres (**CRC**) and the Advanced Livestock Measurement Technologies (**ALMTech**) arrangements in Australia. Each requires investment from multiple cooperating industry partners to leverage substantial government funding and has been rated highly by the industry. A key advantage of these structures is 1) their longevity (5–15 years depending on performance) 2) the development of a powerful heads of agreement collaborative structure that facilitates seamless collaboration across institutions simplifying IP and funding systems and 3) the intense engagement between commercial and academic researchers creating new science-based solutions to the industry’s growing challenges.

In each of the examples above, some international cooperation is possible, but this is generally peripheral. Far greater international collaboration is required to counter the decline in personnel at individual institutions and to create stronger effective teams that have sufficient scale and depth to generate critical new fundamental work and deliver meaningful and applicable outcomes adequately and efficiently. For this to happen, funding structures should be reviewed and adjusted against a global need and dispersed expertise. This demands new thinking in funding arrangements and a reduction in importance placed on IP protection at the expense of timely delivery and the broad application of the results. The industry and government can increase engagement to lead and facilitate this change. New relationships should energize and focus institutional research and educational activity and government can be engaged through the societal and economic value of engagement and investment. Successful new structures have the potential to turbocharge outcomes at scientific and commercial levels with the international interaction of young and senior researchers, extension, and industry people providing an exciting prospect and incentive to consider careers in the production of meat products.

The Innovative Tools for Assessment and Authentication of chicken and beef meat, and dairy products QualiTies (INTAQT) project provides a further example of a multi-actor EU funded research program with 21 partner organizations across 10 countries engaged in measuring a wide range of production-to-consumer measures comparing small scale nonintensive and intensive livestock systems. Again, however this is a single call funding outcome that does not guarantee continuity or long-term ability to advance further from the initial findings.

A current beef genomics project with Irish Cattle Breeding Federation (ICBF), US (USDA), and Australian (MLA) partners evaluating genomic linkages across cattle populations coupled with consumer eating quality evaluation of over 8,000 cattle provides a strong example of global collaboration creating value beyond that possible if pursued individually without combined analysis.

The International Meat Research 3G Foundation (**IMR3GF**) is a further global initiative established from UNECE activity to provide common carcass and consumer evaluation standards and a collaborative not for profit structure to store and share eating quality data for research and commercial application thereby providing scale beyond that affordable by individual organizations acting alone. This offers an attractive nonaligned international collaborative model with support provided by collaborative funding to achieve its full potential.

The disturbing challenge of scientific intimidation around peer-reviewed publication and personal attacks on individuals providing evidence at odds with ideological views as reported by Rubin (2020) and Flegal (2021) must also be strongly resisted, with strength in numbers supporting such works perhaps a means to defray individual attack. Science must stand on the evaluation of evidence rather than ideology or politically correct assertion.

## Conclusions

This paper provides examples of effective research engagement between industry and academic scientists. More are needed and will evolve. Central themes should be engagement with youth from a young age to encourage curiosity, interest, and positive views of meat as a food and the livestock and meat industries that produce it. A further essential element is greater collaboration across traditional boundaries to counter the decline in human and physical capital and to accelerate industry innovation and productivity. Greater collaboration should occur at institutional, country, and global levels which will demand open sharing of resources and expertise. Industry access and early commercial application to improve productivity, human diet, and environmental outcomes supplant the pursuit of controlling intellectual property and related revenue, a return to more open science, and shared resources. A careful study of the issues discussed here reveals that two major changes are required: (1) industry (the beneficiaries of much of the research outcome and need for human resources), needs to become far more involved in investing/funding and (2) to maintain and increase industry’s involvement, scientists should deliver timely solutions to applied as well as basic research challenges.

We hope this document is the beginning of a dialogue and encourages the widespread involvement of those who care about animal agriculture and its societal role to address problems raised here to provide long-lasting solutions before it is too late.

## About the Author(s)

**Rod Polkinghorne** is a highly experienced research team leader who has pioneered the use of untrained consumer sensory evaluation as a primary tool to quantify the eating quality impact of multiple production factors from genomics through on farm management, transport, processing, maturation, value adding, and cooking method on individual beef and lamb muscles. This data, from 11 countries and 200,000 consumers, have been used to build the Meat Standards Australia and other consumer prediction models which underpin grading at a cut level and support strong retail branding. Rod is an adjunct professor at the University of New England and was awarded an honorary doctorate from Murdoch University and an Order of Australia in recognition of services to the beef industry. He has been directly involved in feedlotting, grassland breeding and fattening, beef fabrication and retailing with this strong commercial background assisting in driving application of scientific outcomes at farm, factory, retail, and government level. His work has included strategic industry evaluation and engagement in advancing and advocating for evidence-based decision making relating to animal agriculture’s essential role in human nutrition, environmental and societal structures.

**Mohammad Koohmaraie** joined IEH as the Chief Executive Officer of the Meat Division in 2008. He received his Ph.D. in Meat Science and Muscular Biology from Oregon State University, his M.S. in Meat Science from Texas A&M at Kingsville, and his B.S. in Animal Science from Pahlavi University. Throughout his career, Dr. Koohmaraie has focused his research efforts on the biological mechanisms regulating meat tenderness. Starting with the E. coli O157:H7 outbreak in the Northwest, Dr. Koohmaraie has been heavily involved in leading and conducting research projects to help the meat industry eliminate E. coli O157:H7 from the red meat supply. After 24 years as a USDA scientist, Dr. Koohmaraie left the public sector to pursue his passion in the area of beef safety and quality in the private sector. Throughout his impressive career Dr. Koohmaraie has received numerous awards such as the Agricultural Research Service’s Scientist of the Year Award in 1992 and 2001, the Agricultural Research Service’s technology transfer awards in 2004 and 2008, and the USDA Secretary Award in 1995 and 2001. In 2012, Dr. Koohmaraie was inducted of the Meat Industry Hall of Fame. Dr. Koohmaraie has published over 400 scientific publications and has given over 300 invited talks to U.S. and International Organizations. Dr. Koohmaraie is an ISI Highly Cited Scientist.

**Collette Kaster** currently serves as the Chief Executive Officer of the American Meat Science Association (AMSA) as well as the Executive Director for PAACO, the Professional Animal Auditor Certification Organization. She has 30 years of experience in the meat industry working where she led teams in the areas of quality assurance, technical services and livestock procurement including the implementation of some of the first animal handling and audit programs, along with the implementation of modern handling and CO^2^ stunning equipment. She is an AMSA Fellow and has served on the Advisory Committee for Meat and Poultry Inspection along with many other industry committees and task forces in the areas of animal welfare, food safety, and meat quality.

**Declan Troy** is the Assistant Director of Research, Teagasc—The Irish Agriculture and Development Authority. He is also Director of Technology and Knowledge Transfer for the Teagasc Food Programme and is Head of the Teagasc Ashtown Food Research Centre. During his career, he has published over 150 scientific peer reviewed publications, books, book chapters and scientific articles, mainly in food quality. Declan has always encouraged the up-take of science-based innovations by the food industry and has interacted widely with the sector to this end. His work has contributed to the introduction of innovative technologies at industrial level particularly in Irelands competitive beef sector. In 2017 he was awarded the American Meat Science Association’s International Award. He has collaborated in his research programme with many different research groups from Europe and all around the world including Australia, Korea, New Zealand, Uruguay, China, Brazil, and the USA. Declan has fostered highly successful international collaborations and exchange of knowledge in food science by coordinating EU and nationally funded research projects worth more than €120 million that supported 135 PhD students globally in different laboratories at collaborating institutions. Most recently, he has been appointed as the Director of the National Consumer Food Centre in Teagasc funded by the Department of Agriculture, Food, and the Marine. Declan sits on many national and international committees formulating research priorities in food science and advising state agencies and companies. He is currently a member of the UNECE Working Group on Meat Quality. He was Chairman of the World Congress of Food Science and Technology 2016 in Dublin in his capacity as President of the Institute of Food Science and Technology of Ireland (2016–present). He has also been appointed (2019) as head of the International Secretariat of the International Congress of Meat Science and Technology (ICoMST) of which he was chair both in 2006 (Dublin) and 2017 (Cork) and he is the Academic Leader of Meat Technology Ireland (MTI) a €20m meat industry led research and innovation programme. Recently Declan has been appointed to the Board of Directors of the International Union of Food Science and Technology (2022-2024).

**Andrea Rosati** graduated at University of Perugia (Italy) in Animal Science, then he received a M.Sc. in Animal Genetics and a Ph.D. in Biometry and Animal Genetics both at University of Nebraska-Lincoln (USA). He had worked from 1993 to 2001 for the Italian Animal Breeders Organization (AIA) with growing responsibilities on animal performance recording and genetic evaluation at a national scale. From 2002 to 2004 he had been general manager of the Italian Animal Genetics Laboratory. Later he became the Secretary General of the European Federation of Animal Science (EAAP) and of the World Association for Animal Production (WAAP). He had also managed the International Committee for Animal Recording (ICAR) until 2014 dealing with the world standards for animal identification, performance recording and genetic evaluation. He also coordinated and managed 3 EU international research projects and participated as a partner to 19 other EU projects. He gives lectures in many different countries about animal production, sustainability, and development. He is an author of many scientific articles and co-author of two books. Andrea was the co-founder of four animal science journals and is still part of their management boards. His daily activities are to lead a team organizing scientific conferences, webinars, workshops, scientific journals, international research projects, etc. about animal science.
